# Improving the Tribological Performance of POM through the Incorporation of Bio-Based Materials

**DOI:** 10.3390/polym16162310

**Published:** 2024-08-15

**Authors:** Lucas M. Kneissl, Roberts Joffe, Mitjan Kalin, Nazanin Emami

**Affiliations:** 1 Polymer-Tribology Group, Division of Machine Elements, Department of Engineering Sciences and Mathematics, Luleå University of Technology, Campus Porsön, 971 87 Luleå, Sweden; 2 Laboratory for Tribology and Interface Nanotechnology, Faculty of Mechanical Engineering, University of Ljubljana, Bogišićeva 8, 1000 Ljubljana, Slovenia; 3 Polymeric Composite Materials, Division of Materials Science, Department of Engineering Sciences and Mathematics, Luleå University of Technology, Campus Porsön, 971 87 Luleå, Sweden

**Keywords:** tribology, polymer composites, polyoxymethylene, cellulose fibers, wear resistance

## Abstract

Polyoxymethylene (POM), an engineering polymer commonly used in tribological applications, is often reinforced with fossil-based fibers such as carbon and/or glass fibers to improve its properties. To find more sustainable solutions, in this study, the tribological performance of POM/short cellulose fiber composites at different sliding conditions is investigated. An improvement in the wear coefficient of roughly 69% is observed at the harshest conditions of 5 MPa and 1 m · s^−1^ with only 10 wt.% cellulose fibers. The friction behavior is furthermore stabilized through fiber addition, as the unfilled polymer did not show a steady state. No signs of thermo-oxidative degradation are found after tribological testing. This study presents promising results for sustainable wear-resistant polymer materials in tribological applications.

## 1. Introduction

In tribological applications, the use of polymeric materials is gaining interest for a variety of applications, even in components subjected to high speed and high contact pressure, such as gearboxes, flywheel rotor bearings and others [1,2]. Especially regarding the aspect of weight reduction, polymers can provide benefits over classical engineering materials like metals due to their advantageous strength-to-weight ratio, as well as chemical and corrosion resistance. This in turn also improves the efficiency of machinery, such as an increased driving range of electric vehicles through weight reduction. The ultimate consequences of such improvements include emission reductions, energy savings, multifunctionality and, therefore, monetary benefits [2,3,4,5].

One commonly used thermoplastic polymer for tribological applications is polyoxymethylene (POM) due to its preferable wear resistance, chemical stability, good mechanical properties and self-lubricity [6,7,8]. To improve its properties to meet the requirements of higher-demand applications, it is often reinforced with mostly fibers such as glass or carbon fibers. But these present issues in terms of their sustainability, as they are energy intensive to produce and often expensive [5,9,10]. An affordable alternative can be the use of natural fibers due to their abundance, mostly low cost, lower abrasiveness to processing equipment and beneficial sustainability (e.g., biodegradability, recyclability) [10,11,12].

The tribological evaluation of engineering thermoplastic/natural fiber composites is not necessarily common in the literature. A few examples are the works by Mohanty et al. [13], Nishitani et al. [14] and Hashmi et al. [15]. The first two studies found some beneficial effects of natural fiber incorporation into different matrix polymers for both friction and wear, while the latter obtained a significant increase in the coefficient of friction but an improved specific wear rate through fiber addition into the polymer composite, also allowing for higher loads to be tested.

Furthermore, the specific combination of POM with natural fibers, albeit seemingly intuitive due to the favorable polarity of POM towards the hydrophilic reinforcements, is even more scarce. Deepanraj et al. [16], for example, manufactured POM/flax fiber composites, resulting in a reduction of the wear of the composite under different sliding conditions with increasing fiber loading. Tang et al. [17] used ionic-liquid-treated long cellulose fibers to reinforce POM, leading to a reduction in wear up to a fiber content of 20 vol.%, while the coefficient of friction was slightly elevated over the neat POM. Xiong et al. [18] used woven flax fabric as reinforcement in their study on the abrasive wear performance of POM. No benefit of the fiber addition on the coefficient of friction was observed: the specific wear rate was only improved for two of the tested conditions, obtaining even detrimental effects for two other sets of parameter combinations [18]. Kawaguchi et al. [19] similarly found an increase in specific wear rate upon fiber incorporation. Furthermore, even higher wear rates were found in this study for a POM/glass fiber composite, showing specific wear rates two orders of magnitude higher than for the neat polymer [19]. Zhang et al. [7] also found a major detrimental effect of glass fibers in POM composites for both the coefficient of friction and the specific wear rate at two different p·v values.

The product of the (contact) pressure *p* and the sliding velocity *v* in a tribo-system, i.e., the pressure–velocity limit, p·v, is often used as an indicator for the limit of the operating conditions of tribomaterials. Only few evaluations for POM have been published so far. Examples are the studies by Siddiqui et al. [20] and Gehlen et al. [21]. Siddiqui et al. [20] studied the impact of sliding speed, normal load and countersurface volume on the tribological performance of neat POM, highlighting the importance of heat dissipation within the contact and, therefore, the steel countersurface thickness. The maximum p·v value tested was 4.8 MPa · m · s^−1^, where POM still performed without a significant increase in wear. Gehlen et al. [21] examined the p·v range of POM/carbon black composites through varying the sliding speed at a constant contact pressure of 1.15 MPa. The sliding speed was determined to be limited to 1.7 m · s^−1^ at room temperature, while, beyond this, the specific wear rate significantly increased. The coefficient of friction maintained a stable value over the whole tested range.

The comparison between natural fibers and common fossil-based reinforcements in POM, as mentioned, shows the potential of natural materials as reinforcements and, therefore, an opportunity to replace such traditional material systems with more sustainable options. Since POM and its composites are widely used in tribological applications, especially using fossil-based reinforcements [7,22,23], it is important to evaluate more green alternatives to these materials, which have seldomly been studied [17,24]. Furthermore, investigating the possible use range of such composites is crucial, so as to expand the range of applications of more sustainable engineering polymer materials.

This study provides further insight into the wear mechanisms of cellulose-fiber-reinforced POM composites in dry conditions over a range of different p·v values using a pin-on-disc setup. The mating surfaces were examined in terms of their appearance and chemical composition to facilitate the interpretation of the friction and wear data. To date, no such study for a natural-fiber-reinforced POM composite system has been presented, to the authors’ best knowledge.

It is expected that the incorporation of natural fibers will increase the wear resistance and the use range of POM. This knowledge will prove useful for developing novel thermoplastic (POM) bio-based composites that can be applied in harsh, demanding conditions, adding to the applicability of low-melting-point thermoplastic composites in tribological applications while providing possible alternatives to fossil-based reinforcements that are currently used.

## 2. Materials and Methods

### 2.1. Chemicals

Polyoxymethylene Ultraform N2320 003AT pellets were provided by BASF (BASF SE, Ludwigshafen, Germany). The regenerated cellulose fibers, provided by Cordenka GmbH & Co. KG (Obernburg a. Main, Germany), were Rayon chopped viscose fibers with a density of 1.5 g · cm^−1^, an average length of 2 mm and a diameter of 12–15 μm. The strength of the fibers is 830 MPa and the modulus is 20 GPa, according to the technical specifications provided by the manufacturer [25].

### 2.2. Processing

**Figure 1:**
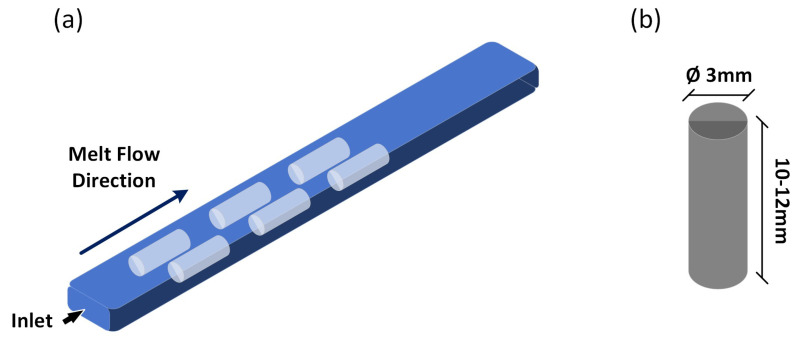
Schematics of the (**a**) injection-molded bars from which the individual tribo-pins were cut and (**b**) geometry of the cylindrical pins used for tribological testing.

The materials were processed by melt-mixing the polymer and fibers for 15 min at 180 °C in a Plastograph Torque Rheometer (Brabender GmbH & Co. KG, Duisburg, Germany) and subsequent injection molding into rectangular bars using a Haake MiniJet (Thermo Fisher Scientific, Dreieich, Germany). The temperatures for injection molding were 190 °C for the cylinder and 85 °C for the mold. Further details on the processing can be found in [26]. Five materials were tested: POM reinforced with 10, 20 or 30 wt.% regenerated cellulose fibers, neat POM directly injection molded using the pellets as received after drying and POM pellets injection molded after being subjected to the melt-mixing procedure to mimic the treatments to which the composites were exposed. The latter is referred to as processed POM in the following, whereas, for the composite samples, the fiber content is used as the descriptor. The samples for tribological testing were then cut from the bars in the direction of the melt flow, as indicated by the light blue cylinders within the bar in Figure 1a.

### 2.3. Characterizations

#### 2.3.1. Tribological Investigations

Tribological experiments were carried out using a CETR UMT 2 (Bruker, Billerica, MA, USA) in linear unidirectional mode and dry conditions. As the countersurface, 100Cr6 steel discs were used. The discs were ground to a surface roughness of 0.15 ± 0.015 μm. The pins were cut to a diameter of 3 mm and a length of ca. 12–15 mm (see Figure 1b). To assess the wear of the materials, both the mass change and the change in the z-coordinate, as measured by the tribometer, were compared in preliminary evaluations, leading to comparable results. Therefore, in the following, only the wear data obtained using the z-coordinate change are presented and discussed. Two different speeds and contact pressures were chosen to cover a range of p·v values from 1.5 to 5 MPa · m · s^−1^. The total sliding distance was kept constant at 20 km. All parameters are listed in Table 1.

#### 2.3.2. Scanning Electron Microscopy

To characterize the surfaces of the pins and steel countersurfaces after tribological testing, a JSM-IT100 and a JSM-IT300 (JEOL Ltd., Tokyo, Japan) scanning electron microscope (SEM) were used. Energy-dispersive X-ray spectroscopy (EDS) was used to investigate the pins and discs regarding possible material transfer. Prior to investigation, all non-conductive samples were sputter-coated to ensure electrical conductivity.

#### 2.3.3. White Light Interferometry

Using a NewView 7300 Scanning White Light Interferometer (Zygo Corporation, Middlefield, CT, USA), the worn pin surfaces were characterized at different magnifications to gain an understanding of the wear mechanisms through the topography of the pins after testing. The data were further processed using MountainsMap®, version 9.3. Furthermore, a White Light Confocal Microscope (HiROX, Tokyo, Japan) was used for investigating the wear tracks on the countersurfaces.

#### 2.3.4. Fourier-Transform Infrared Spectroscopy

To investigate the chemical composition and to evaluate surface chemistry of the pins after tribological characterization, Fourier-transform infrared spectroscopy (FTIR) was performed using a Thermo Scientific Nicolet Summit FTIR spectrometer (Thermo Fisher Scientific, Dreieich, Germany) on the pins’ surfaces prior to and after tribo-testing.

## 3. Results

### 3.1. Tribological Performance

The values reported below for the coefficient of friction and wear coefficient are average values.

#### 3.1.1. Friction

**Figure 2:**
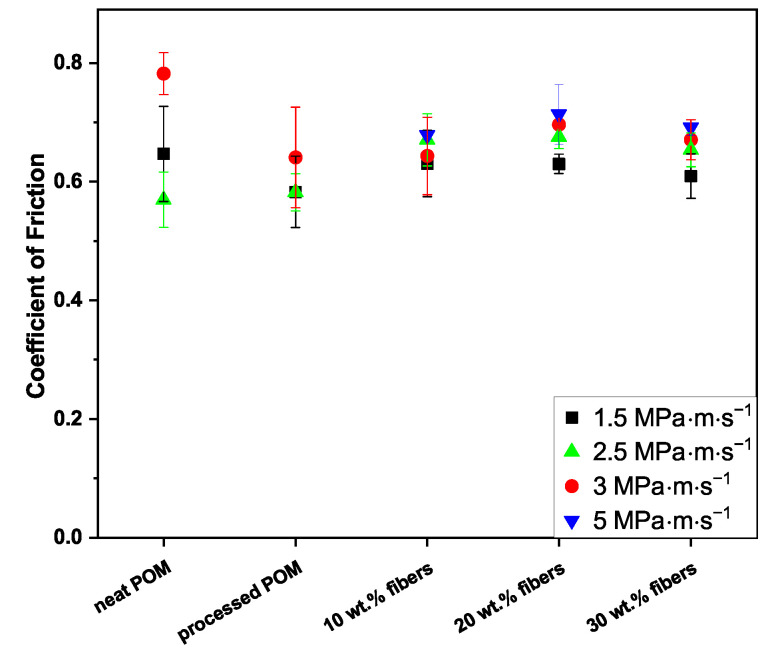
Coefficients of friction of the five tested materials at the four test parameter combinations, represented through the respective p·v values. For p·v=5 MPa · m · s^−1^, no stable friction was obtained for the two unreinforced materials; therefore, the plot shows no data at these positions. The data points depicted are average values. The corresponding experimental conditions can be found in Table 1.

In Figure 2, the values for the coefficient of friction for the four p·v values are plotted against the sample type.

*p* · *v* = 1.5 MPa · m · s^−1^; 3 MPa and 0.5 m · s^−1^

The neat POM and the processed POM present exhibit the highest and lowest coefficients of friction in this dataset, respectively, with values of 0.65 and 0.58. Furthermore, for the composite, the one with 30 wt.% fibers showed the lowest coefficient of friction of 0.61, while the 10 wt.% and 20 wt.% composites both reached 0.63.

*p* · *v* = 2.5 MPa · m · s^−1^; 5 MPa and 0.5 m · s^−1^

For the unreinforced samples, the coefficients of friction lie at 0.57 and 0.58, while 0.67 was obtained for the composites with 10 and 20 wt.%, and a slightly lower value of 0.65 for the 30 wt.% composite.

*p* · *v* = 3 MPa · m · s^−1^; 3 MPa and 1.0 m · s^−1^

The lowest value for the coefficient of friction was found for the processed POM at 0.64, as for the composite containing 10 wt.% fibers. The neat POM showed the highest coefficient of friction of 0.78. The other two composites were in between, with 0.70 and 0.67 for 20 wt.% and 30 wt.% cellulose fiber content, respectively.

*p* · *v* = 5 MPa · m · s^−1^; 5 MPa and 1.0 m · s^−1^

**Figure 3:**
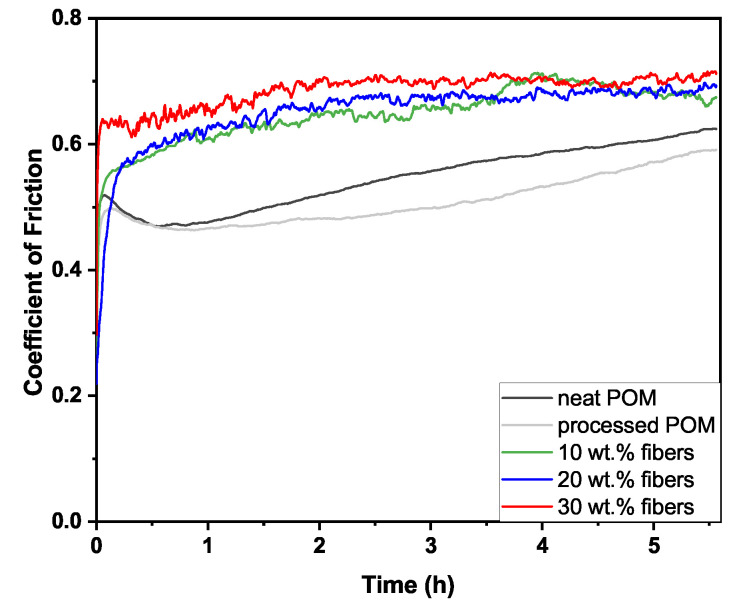
Coefficient of friction as a function of the test time at p·v=5 MPa · m · s^−1^ (contact pressure 5 MPa and sliding speed 1.0 m · s^−1^).

At the highest p·v of 5 MPa · m · s^−1^, no stabilization of the friction curves of the two unfilled POM samples (neat POM, processed POM) was obtained, as clearly observable in Figure 3. Therefore, no steady-state coefficient of friction could be measured under the given test conditions. The 10 wt.% samples (green curve in Figure 3) are around a value of 0.68, though not fully stable over the sliding distance of 20 km. Both the 20 and 30 wt.% samples, on the other hand, show coefficients of friction of 0.71 and 0.69 after stabilization.

#### 3.1.2. Wear

**Figure 4:**
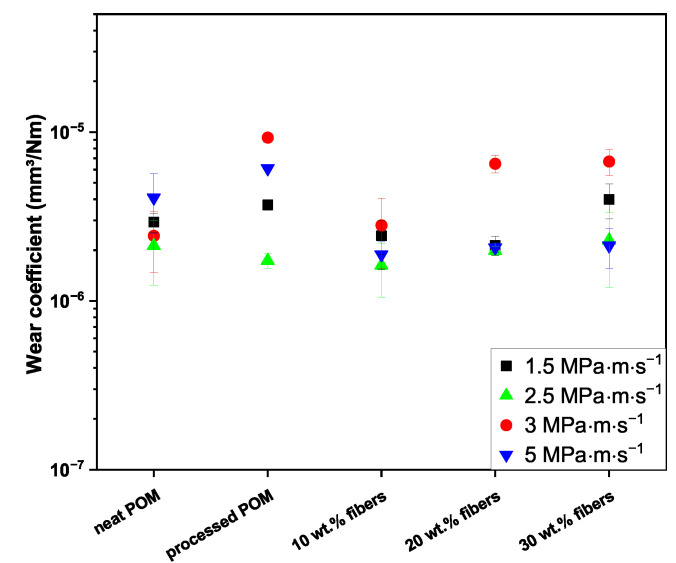
Wear coefficients of the five tested materials at the different p·v values. The data points depicted are average values. The corresponding experimental conditions can be found in Table 1.

The wear coefficients of the five sample types are shown in Figure 4 for the different p·v conditions. To improve readability, the order of magnitude and the unit are omitted in the following section. All values presented are on the order of magnitude of $10^{-6}$; the unit for the wear coefficient is mm^3^ · (N · m)^−1^.

*p* · *v* = 1.5 MPa · m · s^−1^; 3 MPa and 0.5 m · s^−1^

The two types of unreinforced polymer exhibited wear coefficients of 2.93 and 3.70 for the neat and processed POM, respectively. The 20 wt.% composite shows the lowest wear coefficient of 2.13. The composite containing 30 wt.% cellulose fibers has the highest value of 4.00, while the value for the 10 wt.% composite was calculated as 2.43.

*p* · *v* = 2.5 MPa · m · s^−1^; 5 MPa and 0.5 m · s^−1^

The two unfilled samples exhibited wear coefficients of 2.12 and 1.73, while the values for the composites ranged from 1.62 at the minimum for the composite containing 10 wt.% fibers to 2.28 at the maximum for the 30 wt.% composite.

*p* · *v* = 3 MPa · m · s^−1^; 3 MPa and 1.0 m · s^−1^

The neat POM and the composite containing 10 wt.% regenerated cellulose fibers exhibited wear coefficients of 2.43 and 2.81, respectively, while the processed POM showed the highest wear with a coefficient of 9.27. The values for the two remaining composites were calculated to 6.50 and 6.69 for the samples reinforced with 20 and 30 wt.% cellulose fibers.

*p* · *v* = 5 MPa · m · s^−1^; 5 MPa and 1.0 m · s^−1^

The highest wear coefficient at p·v of 5 MPa · m · s^−1^ was obtained from the processed POM samples with a value of 6.10, while a value of 4.08 was determined for the neat POM. The 10 wt.% composite showed the lowest wear coefficient with 1.88, while the values obtained for the 20 wt.% and 30 wt.% composites lie at 2.07 and 2.13, respectively.

### 3.2. Scanning Electron Microscopy

#### 3.2.1. Stainless Steel Disc Countersurfaces

**Figure 5:**
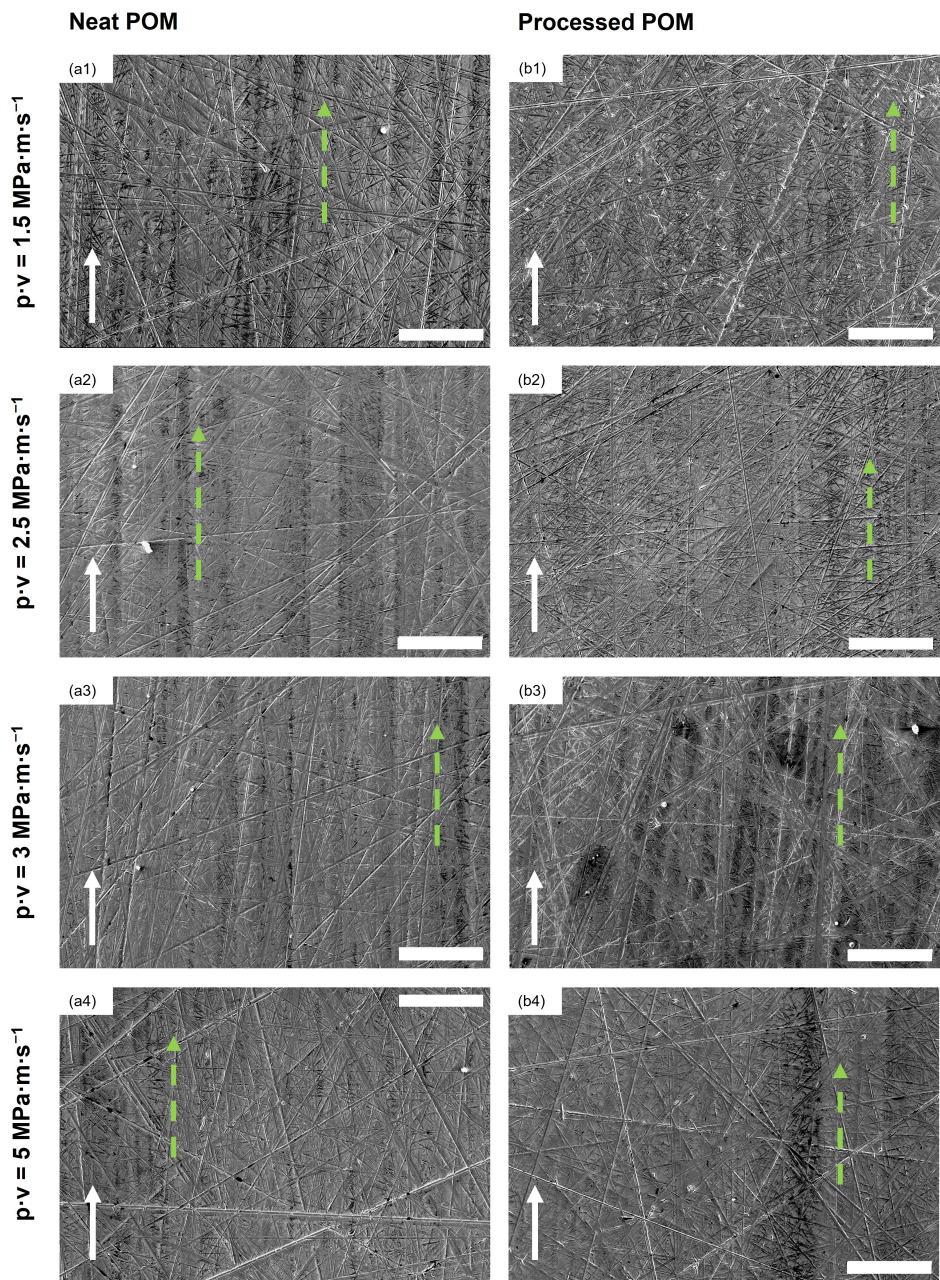
SEM images of discs after tribological testing of neat POM (**a1**–**a4**) and processed POM (**b1**–**b4**) at all different conditions. The white arrows indicate the sliding direction, while the dotted green arrows mark the thin lines of transferred material. All scale bars are 100 μm.

**Figure 6:**
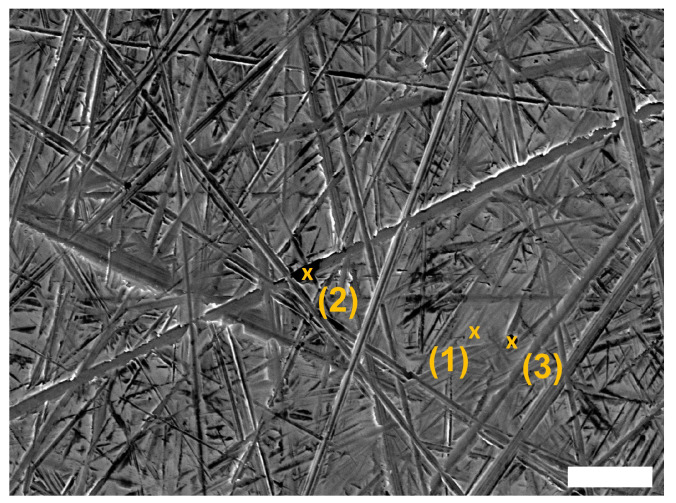
SEM image of countersurface from the testing of neat POM sample with indicators where the spectra were measured. Spot 1: disc surface without tribofilm coverage; Spots 2 and 3: transferred polymeric material. The length of the scale bar is 20 μm.

In Figure 5, the SEM images of stainless steel countersurfaces after tribological testing for the two unreinforced POM samples (neat POM, processed POM) at the four different testing conditions are shown. Within the wear track of the discs, a very thin layer of transferred polymer can be found in long lines parallel to the sliding direction, indicated by the dotted green arrows in the figure. The surface of the track is not fully covered. The scratches on the surface of the disc caused by the roughness preparation are only marginally filled by polymer from the polymer pins; no clear deformation of the asperities of the stainless steel countersurfaces can be seen. The presence of the polymer on the countersurfaces has been confirmed using EDS (cf. Figure 6, Table 2), which was used to detect the carbon content on the surface. This appearance is consistent for all unfilled samples under all p·v conditions. Furthermore, no clear difference in thickness of these layers can be observed in the SEM images for different testing conditions. Therefore, representative SEM images are shown below in Figure 5.

**Figure 7:**
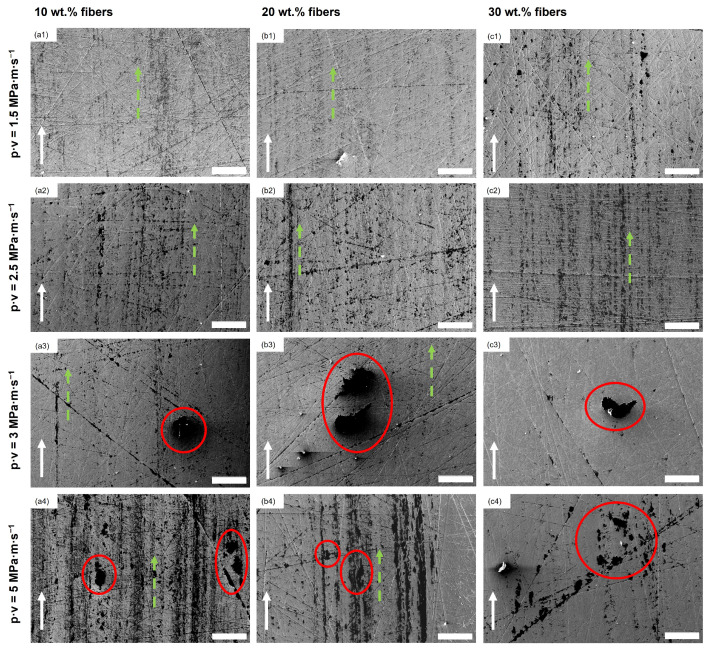
SEM images of discs after tribological testing of the composite materials at all different conditions. (**a1**–**a4**) 10 wt.% fiber composites, (**b1**–**b4**) 20 wt.% fiber composites, (**c1**–**c4**) 30 wt.% fiber composites. The white arrows indicate the sliding direction. The dotted green arrows mark the thin lines of transferred material; the red circles highlight larger, thicker patches. All scale bars are 200 μm.

**Figure 8:**
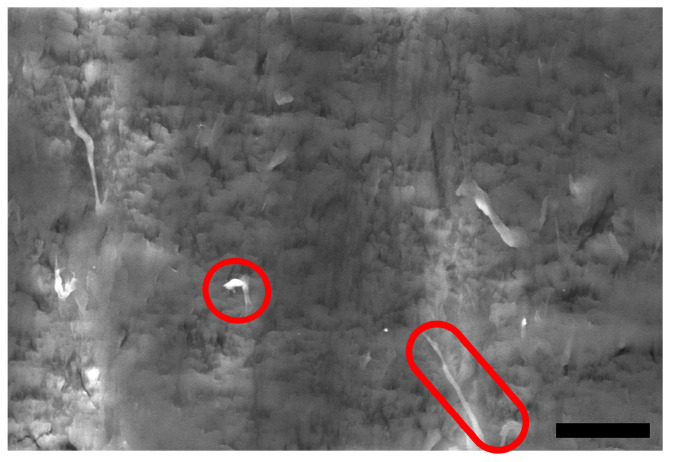
Representative SEM image of a patch of transferred material from a pin with 30 wt.% fibers on the surface of the disc after tribological testing at p·v=3 MPa · m · s^−1^. The red circles highlight the pieces of fibrillated cellulose fibers embedded in the polymer patch on the countersurface. The scale bar is 10 μm.

In Figure 7, SEM images of the stainless steel disc countersurfaces for the POM composite samples are shown. Lines of transferred material along the sliding direction can be found for all shown discs, as indicated by the dotted green arrows, while being less prominent for the discs at p·v=3 MPa · m · s^−1^ (cf. Figure 7(a3,b3,c3)) as well as for the 30 wt.% composite sample disc from the experiment conducted at p·v=3 MPa · m · s^−1^ (cf. Figure 7(c4)). Larger patches of material, aside from the line-like transfer, are found at p·v values of 3 and 5 MPa · m · s^−1^, highlighted by red circles in Figure 7 (a3–c3,a4–c4). The countersurface discs from the tests of the 10 wt.% and 20 wt.% polymer composites at the mildest conditions resemble the look of the ones from the tests of the two reference materials, showing only low amounts of material transferred onto the disc in parallel lines. With the increase in pressure to 5 MPa, the surface coverage increases, with the increase in speed producing long smears of polymer on the surface with a noticeable difference in thickness to the patches found at 5 MPa at the lower speed. The countersurface discs of the tests with composite samples show a presence of patches of transferred polymer, highlighted by the red circles, that are thicker than the very thin layer observed on the countersurface discs from the unreinforced pins. Within these patches, fibrillated cellulose fibers can be found, as shown in Figure 8 as an example from the test of a 30 wt.% sample at p·v=3 MPa · m · s^−1^. EDS was used to identify the fibers. Nevertheless, no loose fiber pieces were found on any of the discs for all tested materials and conditions.

#### 3.2.2. Polymer Pins

**Figure 9:**
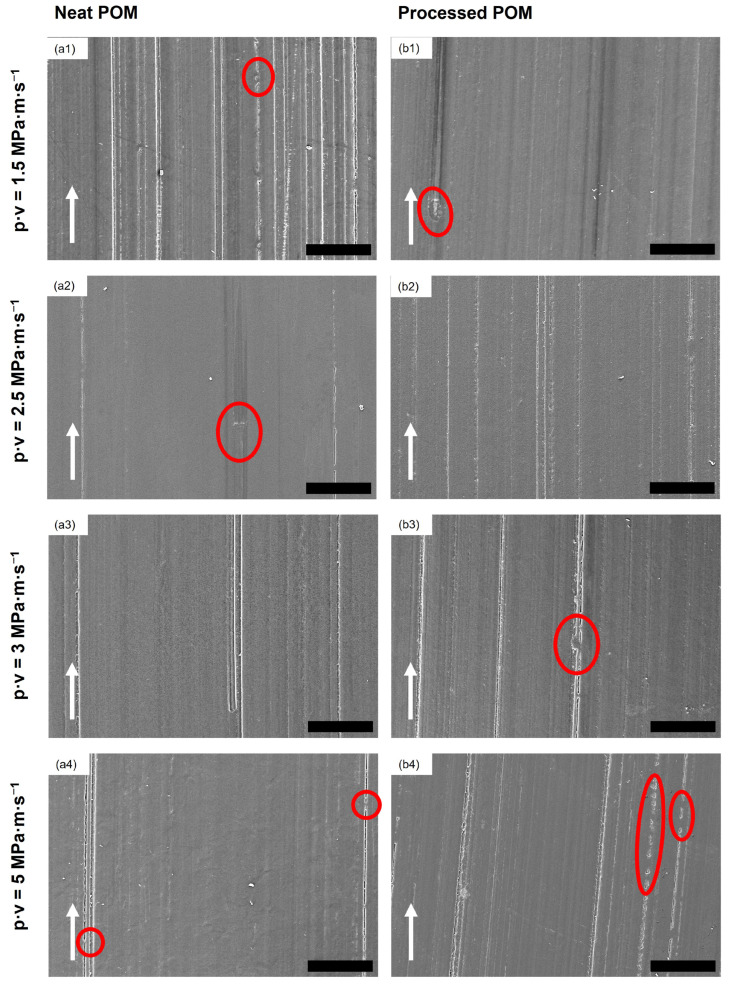
Representative SEM micrographs of pins made from the two reference materials after tribo-testing at the four different parameter variations (given in the row titles). (**a1**–**a4**) neat POM, (**b1**–**b4**) processed POM. The white arrows indicate the sliding direction. The red circles highlight plastically deformed polymer which (in parts) covers scratches on the pin surface. Scale bars are 100 μm.

**Figure 10:**
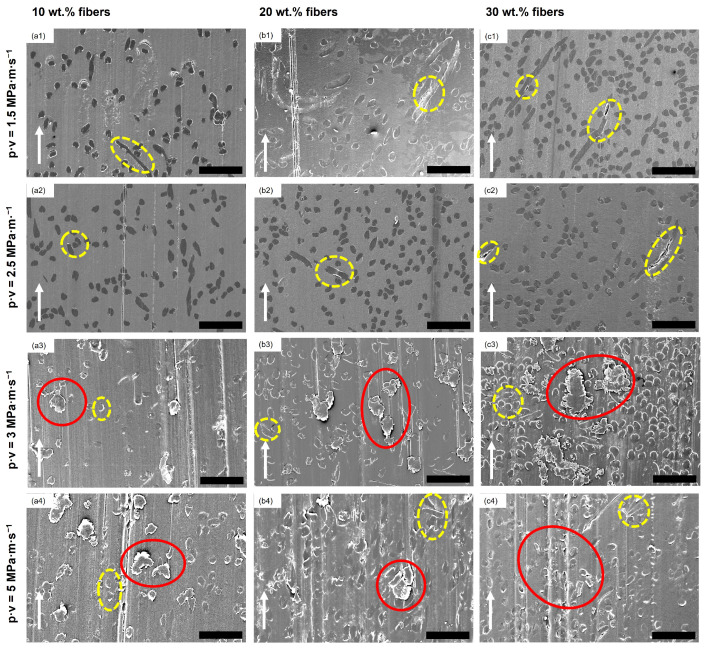
Representative SEM micrographs of pins made from the three composite materials after tribo-testing at the four testing conditions (given in the row titles). (**a1**–**a4**) 10 wt.% fiber composites, (**b1**–**b4**) 20 wt.% fiber composites, (**c1**–**c4**) 30 wt.% fiber composites. The white arrows indicate the sliding direction. The red circles highlight flake-like debris on the surface of the polymer composite pins; the circles with a dotted yellow outline point out regions of fiber debonding. Scale bars are 100 μm.

As seen in Figure 9, grooves and scratches of different depths and widths parallel to the sliding direction are apparent on all of the surfaces of the unreinforced polymer pins. No loose particular debris can be seen on the surface of these pins, but areas where the polymer (partially) covers the scratches and grooves due to plastic deformation, which is highlighted by the red circles in the figure. In Figure 10, the SEM micrographs of the composite pins at the various testing conditions are depicted. Flake-like debris, which has not been re-attached to the pins, is found on the surface of the pins tested at p·v=3 and 5 MPa · m · s^−1^, indicated by the red circles (cf. Figure 10(a3–c3,a4–c4)). In none of the SEM micrographs at any of the tested conditions fiber pullout is observed, while, for all 12 sample sets, some debonding of the fibers from the matrix is seen, as highlighted by the circles with a dotted yellow outline in Figure 10.

**Figure 11:**
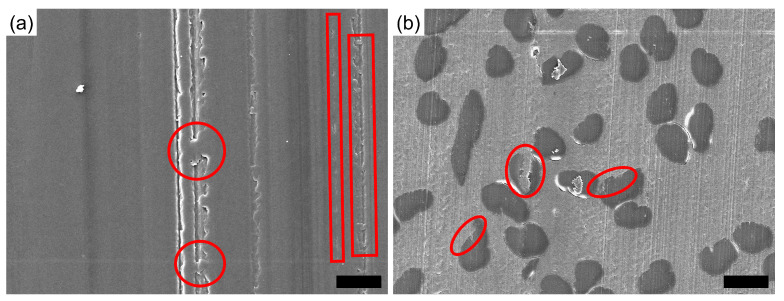
Magnified micrographs of a neat POM (**a**) and a 30 wt.% composite (**b**) pin after tribo-testing with areas of deformed polymer, which has been smeared over the surface, (partially) covering scratches or fibers. These areas are highlighted by the red circles and boxes in the images. Scale bars are 20 μm.

Polymer patches (partially) cover scratches or even fully fill up some of them, as highlighted in Figure 11a, while fibers in the composite pins are also partially covered by matrix material being smeared over them, as shown in Figure 11b. The red elements in the figure point to the areas of the mentioned phenomena.

Scratches with smooth edges are apparent on the surface of the pins tested at p·v values of 3 and 5 MPa · m · s^−1^, which are less prominent for the pins of the other two data sets of p·v of 1.5 and 2.5 MPa · m · s^−1^. No larger damage to the integrity of the composite pins can be seen for any of the shown samples.

### 3.3. White Light Interferometry

**Figure 12:**
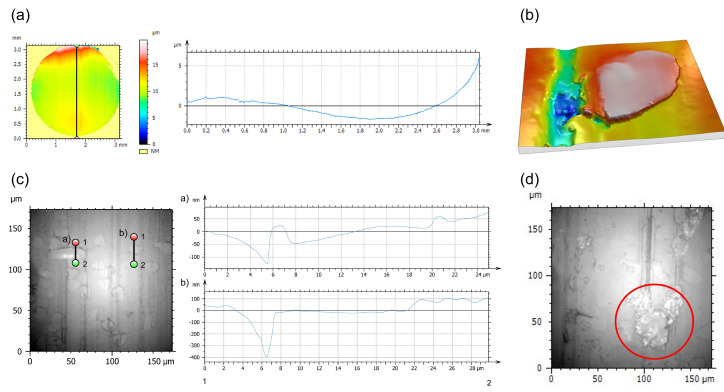
White Light Interferometry data depicting important features on exemplary pin surfaces. (**a**) Exemplary White Light Interferometry image and extracted profile from a processed POM pin, tested at p·v=3 MPa · m · s^−1^. (**b**) 3D rendering of a fiber protruding from the surface of a composite pin after tribological testing. (**c**) Profile measurements of two protruding fibers, highlighting the valley in front of the fibers when approaching in the sliding direction. The measurement is carried out in the direction from 1 (red marker) to 2 (green marker) for the two positions. (**d**) Exemplary image of a patch (highlighted by the red circle) of polymeric material on the surface of a composite pin with 20 wt.% fibers.

A clear wave shape of the pins after tribo-testing was observed for all five material sets, regardless of the conditions of the tribological experiments. An example is shown in Figure 12a. The shape becomes less pronounced with increased fiber content at all p·v values.

All pins show similar surface features, such as grooves parallel to the sliding direction, with similar depths and no obvious differences in overall appearance. The pins made from the composite material showed no obvious signs of fiber pull out. Furthermore, fibers were protruding from the surface of the composite pins. Below, a 3D image can be seen of one fiber clearly being exposed (cf. Figure 12b).

Another feature that could be observed for all composite materials, independent of the testing conditions, was a valley just in front of the fibers when approaching in the sliding direction, as seen in Figure 12c. The depth of these valleys seemingly does not change with different testing conditions or fiber content.

Smaller flake-like patches of matrix material could be found on all samples, smearing over scratches and fibers, as shown, for example, in Figure 12d.

**Figure 13:**
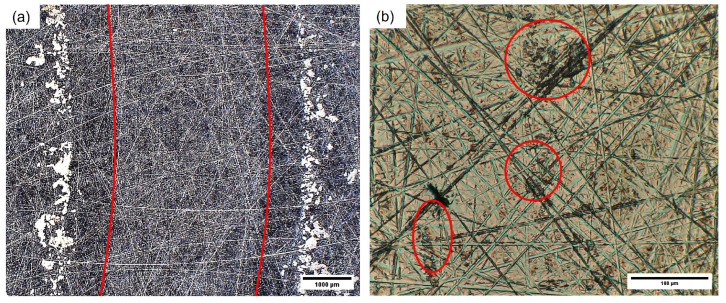
Images of the countersurfaces after tribo-testing. (**a**) Overview image of an example of a wear track with the shape of the track indicated by the red lines. (**b**) Patches of material (highlighted by the red circles) on the countersurface for one exemplary steel disc.

The wear track on the discs is clearly identifiable from images taken with the confocal microscope (see Figure 13a). Flake-like debris is found next to the wear track as well as patches of material attached on the surface of the discs of the composite samples, as observed in Figure 13b.

### 3.4. Fourier-Transform Infrared Spectroscopy

The FTIR data for all curves were normalized against the peak at 1470 cm^−1^, as this peak, representing the methylene bending, does not change except under extreme thermo-oxidative conditions, as described by Vila Ramirez et al. [27].

**Figure 14:**
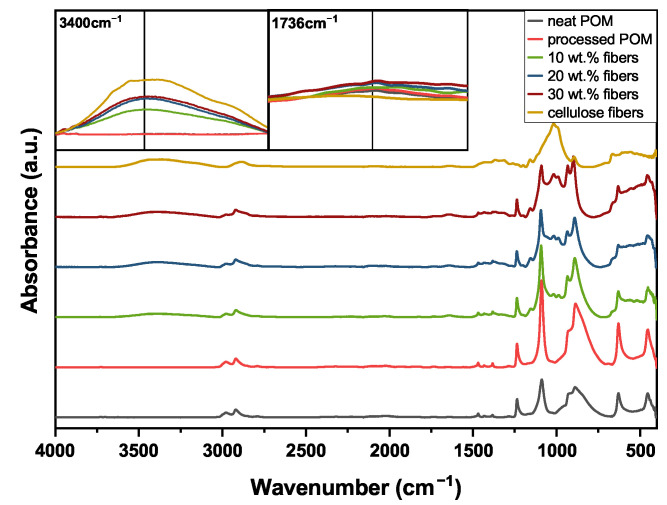
FTIR spectra of the five different sample types prior to testing. The spectrum of the cellulose fibers is added as reference. The two inlets show the regions of interest regarding wavenumbers 3400 cm^−1^ and 1736 cm^−1^.

In Figure 14, an overview over the FTIR spectra of the five different tested material types over the full wavenumber range of 4000–400 cm^−1^ is shown, together with two inserts that highlight the specific regions of interest. For reference, the spectrum for the cellulose fibers is given as well. The spectrum of the fibers shows significant peaks at 3000–3650, 2900 and 1020 cm^−1^, which, respectively, correspond to −OH stretching, −CH bond stretching and C−O stretching. Furthermore, distinct peaks at 1162 (C−O−C asymmetric stretching) and 893 cm^−1^ ($C_{1}$ group frequency) are observed. In general, with increasing fiber amount, a developing wide absorbance band for intramolecular −OH stretching can be found in the range of 3000–3650 cm^−1^ for the composites, as seen in the left insert in Figure 14. With the addition of cellulose fibers, a broadening of the −CH bond stretching peaks at 2920 and less pronounced at 2970 cm^−1^ is identified. Also, an evolution of peaks at 1020 cm^−1^ corresponding to C−O stretching vibrations can be observed for the composites.

At 1736 cm^−1^, no change in the characteristic peak for carbonyl groups can be observed, as is obvious in the right insert in Figure 14. Within one set of sample type, the testing conditions all yielded fairly similar absorption spectra with no significant difference between the p·v variations; therefore, only the two extremes of p·v=1.5 and 5 MPa · m · s^−1^ are plotted together with the signals for the samples prior to tribo-testing.

**Figure 15:**
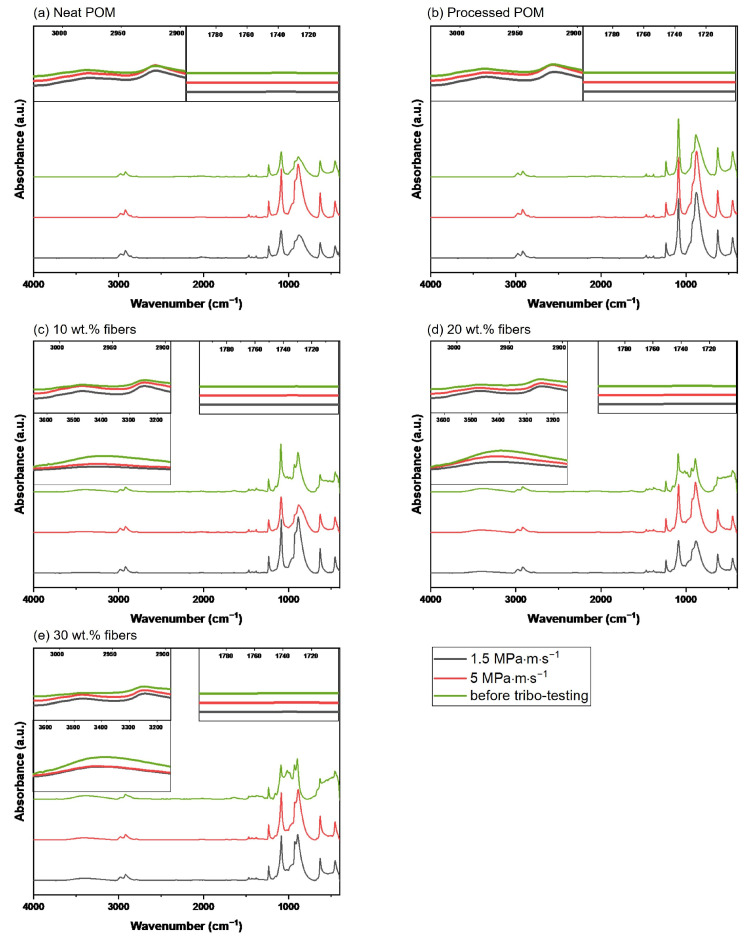
FTIR spectra of (**a**) the neat POM, (**b**) processed POM and (**c**) 10 wt.%, (**d**) 20 wt.% and (**e**) 30 wt.% fiber composites before and after testing. The legend in the bottom right corner is valid for all five graphs, indicating the p·v conditions at which the tribological experiments were conducted. The inserts in the individual graphs refer to the 2 or 3 respective specific wavenumber ranges (2900–2990 cm^−1^, 1700–1800 cm^−1^, 3000–3650 cm^−1^ for the composites in (**c**–**e**), with the latter being neglected for the unreinforced samples.

For all tested composites and under all conditions, the signal at around 3400 cm^−1^ is lower than for the untested composites, as seen in the left bottom insert in Figure 15c–e. Furthermore, the −CH stretching peaks at 2920 and 2970 cm^−1^ show the exact opposite behavior as the intensity increases for the samples after testing, as is obvious in the top left insert in Figure 15c–e. No significant changes for this peak are observed for the two unreinforced materials neat POM and processed POM (cf. Figure 15a,b). For none of the five materials any change at 1736 cm^−1^ is observed, as seen in the right insert in Figure 15a–e.

## 4. Discussion

### 4.1. Tribological Investigations

The findings of the SEM images are not discussed separately, but are part of the discussion in the section below.

**Figure 16:**
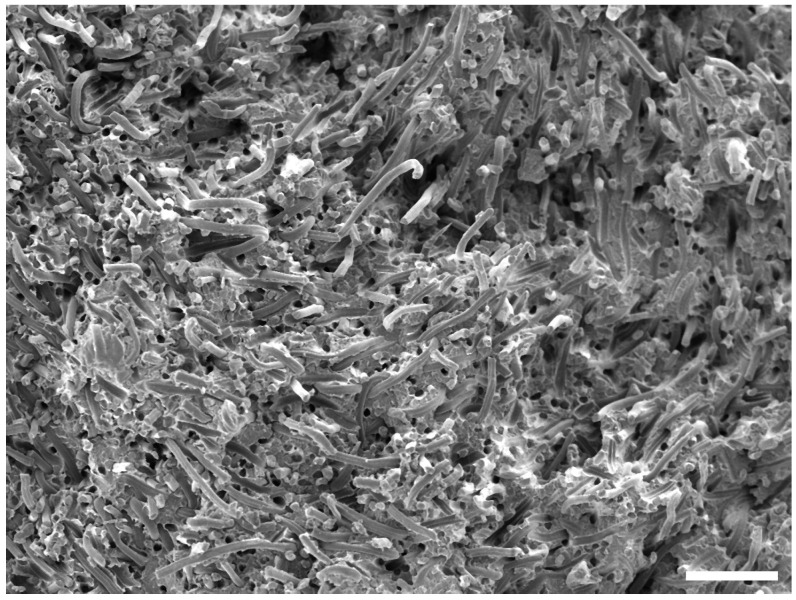
Representative fracture surface image of a composite containing 30 wt.% fibers. Scale bar is 150 µm.

It is important to note that the vast majority of fibers are oriented normally to the sliding direction, as extensive research has proven the influence of fiber orientation on the outcome of tribological studies (e.g., [28,29,30,31]). In a previous work by the authors, the fiber distribution homogeneity and the reason for the fiber orientation has been discussed [26]. A fracture surface image of a 30 wt.% sample is shown in Figure 16 to further support this.

#### 4.1.1. Friction

The observed decrease in the coefficient of friction for the neat POM sample when comparing p·v=1.5 and 2.5 MPa · m · s^−1^ is in line with the findings of Samyn and De Baets, who also found a decrease in the dynamic coefficient of friction of neat POM upon increasing the contact pressure at a constant sliding speed [32]. Similarly, Xiong et al. [18], as well as Zhang et al. [7] in their works, obtained lower coefficients of friction of unreinforced POM when increasing the contact pressure. At p·v=1.5 MPa · m · s^−1^, no significant change in the coefficient of friction is found upon fiber incorporation, opposite to the other data set at the same speed, p·v=2.5 MPa · m · s^−1^. Here, an increase in the coefficient of friction is found when adding regenerated cellulose fibers, while the trend of a decrease in the coefficient of friction with higher fiber content is similar (see Figure 2). A comparable effect was found by Hashmi et al. upon the incorporation of cotton fibers into a polyester matrix, which led to a significant increase in the coefficient of friction [15]. Bajpai et al. also observed that, at low loads and sliding speeds, fiber incorporation did not significantly change the level of the coefficient of friction in their study on poly lactic acid composites [33].

Furthermore, the overall level of the coefficient of friction for the composite samples is higher at p·v=2.5 MPa · m · s^−1^, as the pressure increase from 3 MPa to 5 MPa leads to an increase in interaction between the soft cellulose fiber and, therefore, additional resistance to the sliding movement as the asperities of the steel discs interact with the fiber strands, causing fibrillation of the cellulose fibers (see Figure 8). This is in line with the findings by Tang et al. [17] and Deepanraj et al. [16], who obtained increased friction values when increasing the contact pressure in their studies.

Similar to the findings in the previous study [26], the slight decrease in the coefficient of friction with the increase in fiber loading could be explained through the larger amount of surface covered on the countersurfaces by polymer, as seen in the SEM images of the stainless steel countersurface discs in Figure 7(a1–c1) versus Figure 7(a2–c2), reducing the effective surface roughness of the countersurfaces and, therefore, the mechanical resistance caused by the interaction of surface asperities. This increase in surface coverage is caused by the higher pressure, as compaction of wear debris onto the countersurface is more pronounced.

Contrary to the findings for p·v=1.5 and 2.5 MPa · m · s^−1^ at a sliding speed of 1 m · s^−1^ (p·v=3,5 MPa · m · s^−1^), the coefficient of friction for composite samples is lowest at 10 wt.% regenerated cellulose fibers at both contact pressures (0.64, 0.68) and increases with the further addition of fibers. An increase in fiber content in turn increases the aforementioned interactions between steel disc asperities and fibers and, subsequently, also the coefficient of friction. Tang et al. also found an increase in the coefficient of friction in their study when increasing the fiber loading up to 30 wt.% for their POM/cellulose fiber composites using a twin disc setup [17]. The obtained coefficient of friction at p·v=3 MPa · m · s^−1^ is lower than comparable work by Zhang et al. for their POM composite containing 10 wt.% short glass fibers (>0.8) [7]. The speed increase additionally leads to an increasingly inhomogeneous, patchy structure of the transfer layer on the countersurfaces, clearly observable when comparing, e.g., Figure 7(a1–c1) with Figure 7(a3–c3). The higher speed inhibits the formation of the transfer film through the continuous removal of the deposited polymer and reformation, reducing the homogeneity and overall surface coverage of the tribofilm. This effect is more pronounced at higher fiber content, as the fibers contribute to this removal process due to their normal orientation with respect to the sliding direction. Both the higher tribofilm surface coverage and lower amount of possible mechanical interaction between the fibers and asperities of the countersurface for the 10 wt.% composite are factors for it outperforming the other materials. This has also been reported by Gehlen et al., who found the same effect of rising sliding speeds on the morphology of the transfer layer on the countersurfaces [21].

As an overall trend for the composite samples, and, partially, the unreinforced materials as well, an increase in the coefficient of friction can be observed with increasing harshness of the testing conditions. Due to the higher contact pressure and sliding speed, a higher energy input into the system leads to an increase in apparent contact temperature and, therefore thermal softening, as described, for example, by Samyn and De Baets [32], Yamaguchi and Kashiwagi [34] and Chin and Yousif [31]. This in turn leads to an increased indentation depth of the asperities of the countersurface into the pin surface [32,35]. Nevertheless, when comparing the sample sets at the same contact pressures (p·v of 1.5 vs. 3 MPa · m · s^−1^ and 2.5 vs. 5 MPa · m · s^−1^), an increase in sliding speed from 0.5 to 1.0 m · s^−1^ leads to an increase in the coefficient of friction for all tested samples.

While no stable coefficient of friction for the unreinforced samples was obtainable over the test duration at the harshest conditions, i.e., p·v=5 MPa · m · s^−1^ (cf. Figure 3), the presence of fibers stabilizes the contact, even though at very high friction levels, with the most stable being the composite with the highest fiber loading of 30 wt.% (coefficient of friction of 0.69). In part, this might be explained through the decrease in matrix material present at the interface, which undergoes plastic deformation, as the fibers protruding the pin surface create almost a shielding effect, as seen in the White Light Interferometer and SEM data (cf. Figure 12b and Figure 10(a4–c4)).

#### 4.1.2. Wear

Examining the effect of the applied pressure, it is clear that, for both speeds, the increase in loading decreases the wear coefficient for all tested samples, with the exception of neat POM (see Figure 4). One explanation for this is an increased surface coverage of the steel countersurfaces with transferred material, most prominent for the higher sliding speed of 1 m · s^−1^ (see Figure 7(a3–c3) vs. Figure 7(a4–c4)). The tribofilm covers the asperities of the steel disc, lowering the abrasive wear. This is in line with the findings of Yousif and El-Tayeb, who found a similar behavior for polyester composites reinforced with oil palm fibers at three different loads and sliding speeds [36]. Xiong et al. also obtained a decrease for their tested POM/flax weave composites when increasing the contact pressure, and also found an exception from this trend for the unreinforced POM, which had a significantly increased wear rate at the harshest contact conditions tested [18]. At the higher speed of 1.0 m · s^−1^, the differences, especially for the composites, are more significant than at 0.5 m · s^−1^, especially for the 20 and 30 wt.% fiber composites. The harsher conditions lead to a significant reduction in the wear coefficient, which is the largest in the sample set (see Figure 4). At the lower contact pressure of 3 MPa, the increase in the fiber content contributes to an aggravated removal of transfer film on the steel countersurface (Figure 7(a1–c1,a3–c3)), therefore increasing the abrasive component of the pin wear caused by the countersurface asperities. This is further exaggerated by an increase in speed, which causes for all samples a worsened wear behavior with the exception of neat POM. The effect of the higher velocity as well as the fiber loading is almost negligible at 5 MPa for the composite samples, while, on the other hand, the wear coefficient of the neat and processed POM samples significantly increases (see Figure 4). At this contact pressure, the removal of wear debris from the contact area is hindered through the higher compaction force, thereby mitigating the detrimental effect of increased fiber content, as seen in the SEM images of the steel countersurfaces of p·v=3 MPa · m · s^−1^ (cf. Figure 7(a2–c2)). The most obvious beneficial effect of fiber incorporation is achieved at the two p·v levels of 3 and 5 MPa · m · s^−1^, obtaining a reduction of ca. 69% at both conditions when comparing the best-performing 10 wt.% composite to the processed POM.

The main mechanism of wear for the unreinforced materials is identified as abrasive, as observable from the SEM micrographs of the polymer pins in Figure 9, which show mainly scratches in sliding direction. Similar pin morphology was found in the work by Siddiqui et al. [20] or Unal et al. [37], with grooves caused by cutting and ploughing actions of the disc asperities. The described formation of the lines of very thin, discontinuous transfer film on the stainless steel countersurfaces (cf. Figure 7) was found in different studies—all for tribological experiments in dry conditions—such as by Zsidai et al. [35] or Mergler et al. [38]. The latter study also describes an appearance of the POM pins after testing that is in line with the findings of this present study. Odi-Owei and Schipper [39], on the other hand, could not identify any transferred material from the unreinforced POM pins on the steel discs at any tested speed. This is also an indication of a minor contribution of adhesive action, as only a coverage of the asperities and scratches of the countersurface discs was obvious.

Abrasive wear was also the dominating mechanism for the polymer composite pins (see Figure 10). Cutting and ploughing by the countersurface asperities cause scratches on all pin surfaces and the formation of flaky debris, as visible, for example, in Figure 10(a3–c3) or Figure 10(a4–c4), as well as Figure 12d. The presence of this debris is mainly observed for the samples tested at 1 m · s^−1^, as the higher velocity prevents the adhesion of the polymeric material to the steel countersurface. This is most pronounced when comparing p·v=1.5 and 3 MPa · m · s^−1^, as described before. This, in turn, is detrimental for the wear coefficients of the composite materials.

No fiber pullout was observed for any of the composites and tested conditions; only fibrillation of the fibers through abrasive wear (see Figure 8) was observed, as also described in the previous study [26]. The normal orientation of the fibers with respect to the sliding direction prevents the pullout of larger fiber pieces, as also described by Milosevic et al. [9].

The observations regarding the surface morphology of the pins are in accordance with the findings of Siddiqui et al. [20] and Gehlen et al. [21]. The first study investigated the influence of sliding speed and load on unfilled POM materials, obtaining an increase in wear with an increase in speed at three tested constant loads [20]. The latter observed that, with higher speeds, an increase in the amount of transferred material on the steel discs was obtained over almost the whole range of applied sliding speeds [21]. The lumpy transfer film formed by polymer matrix and fiber debris for p·v=3 and 5 MPa · m · s^−1^ on the countersurface discs of the composite samples (cf. Figure 7(a3–c3,a4–c4)), together with the scratches and valleys on the pin surfaces of all samples, indicates a combination of both adhesive and abrasive wear at these conditions, as adhesive wear gains importance with increasing contact severity [40].

Tang et al. [17] found in their study a significant increase in the wear of the composites with an increase in fiber loading when comparing pristine and cellulose-fiber-reinforced POM. This is contrary to almost all data sets present here, other than the results from the p·v=3 MPa · m · s^−1^, where the incorporation of fibers either had no significant effect or even improved the performance, as is the case for the harshest conditions. Similarly to this, Kawaguchi et al. obtained an increase in specific wear rate for increasing fiber content; all tested composites, however, outperformed the reference composite filled with glass fibers [19].

When comparing to the figure on the comparison of the findings of different publications regarding the wear coefficient of POM sliding against steel shown in the work of Siddiqui et al., the material present within this study clearly shows an improvement versus the data presented. Especially at the highest p·v of 5 MPa · m · s^−1^, the wear coefficient is almost a magnitude lower than the results presented in their study, showing a significantly beneficial effect of the addition of cellulose fibers at all three different loadings [20]. The composites presented in this work further outperform similar materials manufactured and tested by Kawaguchi et al. [19], Xiong et al. [18] or Thirumalvalavan et al. [41] while being subjected to equal or harsher contact conditions. Zhang et al. [7] tested different classic fossil-based reinforcements to improve the tribological performance, among others glass and carbon fibers. The authors found a significant increase in the wear coefficient especially for the incorporation of glass fibers, matching the previously mentioned findings by Kawaguchi et al. regarding a significant increase in specific wear rate of POM [19].

### 4.2. White Light Interferometry

The general surface appearance for all unreinforced samples does not change over all tested conditions, as a constant surface roughness of the stainless steel countersurfaces was chosen. The asperities from the countersurfaces cause the scratches and grooves on the pin surface, while plastic deformation led to a smoothening of the surface, covering parts of the grooves and valleys, as shown in Figure 11, while creating the described wave shape of the pin surface (cf. Figure 12a). The presence of the fibers within the matrix clearly influences the shape of the surface, as the wave shape of the whole surface is less pronounced in the samples reinforced with regenerated cellulose fibers. Therefore, the fibers serve a protective purpose, reducing the amount of matrix deformation and removal during tribo-testing. The wave shape of the samples after the tests clearly indicates a plastic deformation of the pins during testing, as, otherwise, a homogeneous material removal should be expected. The wave shape being less pronounced for the composite materials is explained through the mechanical strengthening in both tensile and flexural loadings, as found in the study previously published by the authors [26] and, therefore, limiting plastic deformation during sliding.

The valleys in front of the fibers (in the sliding direction) were created through debonding and plastic deformation of the fibers during tribo-testing, removing/plastically deforming the matrix material in front of them, while, after the test, regaining their initial positions. This irregular wearing of the fibers can also be observed in the SEM images, where the initially circular fiber cross-sections appear elliptical after testing (cf. e.g., Figure 10(c1) or Figure 10(a2)). Similar findings of such a change in fiber cross-section geometry through deformation and subsequent wear have been reported by Tewari and Bijwe [42] and Yousif and El-Tayeb [36]. Furthermore, as described by Tang et al., an air gap created by the protruding fibers, as shown in Figure 12b between the polymer matrix surface and the steel disc could act beneficially for dissipating frictional heat, leading to a reduction in the wear coefficient through reducing thermal softening [17].

### 4.3. Fourier-Transform Infrared Spectroscopy

The characterization of the cellulose fibers implies that they are indeed viscose fibers, as they are mainly composed of cellulose II, as suggested by the presence of characteristic peaks at 1162 cm^−1^ (C−O−C asymmetric stretching) and 893 cm^−1^ ($C_{1}$ group frequency) [43,44]. The wide absorbance band of the intramolecular −OH stretching in the range of 3000–3650 cm^−1^ is caused by the hydroxyl groups of the cellulose fibers [43,44] (cf. Figure 14). Even though Pang et al. [45] mentioned in their work that POM can show a peak in this region as well, especially in the outermost layers of a sample, no indication of such an absorption band can be found in the unreinforced materials present here. The fiber addition further causes the evolution of the C−O stretching peaks at 1020 cm^−1^ and the broadening of the asymmetrical −CH_2_− stretching at 2920 cm^−1^, as the −CH stretching peak of the pure fibers at 2900 cm^−1^ overlaps with this signal [43,44,46,47]. The lowering of the −OH stretching peak after tribo-testing can be explained by the polymer being smeared over the fiber surfaces, thereby decreasing the signal intensity, as seen in the left inserts in Figure 15c–e. Additionally, the −CH_2_− asymmetric stretching signals of the POM backbone [45,48] at 2920 cm^−1^ and 2970 cm^−1^ increase after testing (see top left inserts in Figure 15c–e), which further indicates a higher amount of polymer present on the surface of the pins. This is in accordance with the SEM images seen in Figure 11b, Figure 10(b3) or Figure 10(c3), where patches of polymer were found on top of fiber areas as well as fibers covered by plastically deformed matrix material.

In contrast to this finding is the result from the White Light Interferometry, where a clear height difference between fibers and matrix was observed (see Figure 12b): the fibers protruding from the surface should decrease the overall contact area between polymer matrix and FTIR detector crystal. This is most likely mitigated by the experimental setup of the FTIR measurements as the pins are brought into direct contact with the attenuated total reflection detector by exerting a normal force onto the pins. No thermal degeneration is observed for any sample after tribo-testing, which would be indicated by an intensity increase at the characteristic carbonyl peak at 1736 cm^−1^ due to carbonyl group formation through thermo-oxidative chain scission [27,45]. Therefore, even at the highest p·v=5 MPa · m · s^−1^, the matrix and fibers withstand the heat generated in the contact without degradation. This is supported by the findings of Siddiqui et al., who investigated the temperature evolution in a POM/steel contact with variations of load, speed and countersurface disc thickness in order to evaluate the importance of heat transfer out of the tribological contact [20]. Their results showed a temperature increase up to roughly 45 °C, which is far below the manufacturer’s recommended maximum usage temperature and below the temperatures of other studies, such as the investigation of Gehlen et al., who conducted experiments at even 85 °C [20,21,49].

## 5. Conclusions

In this study, a thorough investigation of the tribological performance of regenerated-cellulose-fiber-reinforced POM composites at four p·v conditions was conducted. Pin-on-disc tests were performed to examine the impact of the sliding speed and contact pressure as well as the effect of the fiber content.

At the lower contact pressure of 3 MPa (p·v=1.5and3 MPa · m · s^−1^), the coefficient of friction was not influenced significantly through the reinforcement with the regenerated cellulose fibers, as the composites showed even slight improvements, especially at 10 and 20 wt.% over the neat POM. At the higher contact pressure of 5 MPa and the sliding speed of 0.5 m · s^−1^ (p·v of 2.5 MPa · m · s^−1^), on the other hand, the fiber addition led to an increase over the level of the unreinforced samples. No stable friction behavior was obtained for the neat and processed POM samples at the highest p·v value of 5 MPa · m · s^−1^ over the full 20 km sliding distance. Through the addition of the regenerated cellulose fibers, a stabilization of the friction was achieved, effectively increasing the p·v range of POM as a tribo-material. No fiber pullout was found while identifying only few instances of fibers debonding from the matrix, indicating a stable composite. Furthermore, as shown by FTIR, no degradation of the materials was observable. This, in combination with improved wear behavior for the composites over the unreinforced materials in almost all tested conditions, especially the 10 wt.% composite outperforming or at least equaling both unreinforced materials, is a promising result for such composites. The most significant improvements of the wear behavior are found at the p·v of 5 MPa · m · s^−1^, where all three fiber percentages showed reductions by up to 69% vs. the processed POM. Overall, it is important to note that the performance of these composites is reliant on the contact conditions, as demonstrated above. This needs to be considered when designing and selecting certain materials for tribological components. Nevertheless, this work shows that an increased use range of these materials is a prospect in the future, expanding the use of sustainable polymeric composites in more demanding tribological applications and possibly replacing fossil-based reinforcements.

## Figures and Tables

**Table 1:** Parameters for tribo-testing.

Sliding Speed m·s^−1^	Contact Pressure MPa	*p* · *v* MPa·m·s^−1^
0.5	3	1.5
0.5	5	2.5
1.0	3	3
1.0	5	5

**Table 2:** Results of EDS measurements at three selected spots, indicated in the corresponding SEM image (cf. Figure 6). Spot 1 is at a place without polymer covering; spots 2 and 3 are on transferred polymeric material.

Element Spot Number	Fe %	O %	C %	Mg %	Al %	Si %	Cr %
1	92.6		5.8			0.2	1.4
2	81.4	7.7	6.8	0.9		1.8	1.4
3	38.6	26.0	8.8	8.7	17.2		0.7

## Data Availability

The original contributions presented in the study are included in the article; further inquiries can be directed to the corresponding authors.
